# Promise of Fully Integrated PET/MRI: Noninvasive Clinical Quantification of Cerebral Glucose Metabolism

**DOI:** 10.2967/jnumed.119.229567

**Published:** 2020-02

**Authors:** Lalith Kumar Shiyam Sundar, Otto Muzik, Lucas Rischka, Andreas Hahn, Rupert Lanzenberger, Marius Hienert, Eva-Maria Klebermass, Martin Bauer, Ivo Rausch, Ekaterina Pataraia, Tatjana Traub-Weidinger, Thomas Beyer

**Affiliations:** 1 QIMP Team, Center for Medical Physics and Biomedical Engineering, Medical University of Vienna, Vienna, Austria; 2 Department of Pediatrics, Children’s Hospital of Michigan, The Detroit Medical Center, Wayne State University School of Medicine, Detroit, Michigan; 3 Department of Psychiatry and Psychotherapy, Medical University of Vienna, Vienna, Austria; 4 Department of Clinical Pharmacology, Department of Psychiatry and Psychotherapy, Medical University of Vienna, Vienna, Austria; and; 5 Department of Neurology, Medical University of Vienna, Vienna, Austria

**Keywords:** ^18^F-FDG, PET/MRI, image-derived input function, absolute quantification, fully automatic pipeline, Patlak analysis, brain imaging

## Abstract

We describe a fully automated processing pipeline to support the noninvasive absolute quantification of the cerebral metabolic rate for glucose (CMRGlc) in a clinical setting. This pipeline takes advantage of “anatometabolic” information associated with fully integrated PET/MRI. **Methods:** Ten healthy volunteers (5 men and /5 women; 27 ± 7 y old; 70 ± 10 kg) underwent a test-retest ^18^F-FDG PET/MRI examination of the brain. The imaging protocol consisted of a 60-min PET list-mode acquisition with parallel MRI acquisitions, including 3-dimensional time-of-flight MR angiography, MRI navigators, and a T1-weighted MRI scan. State-of-the-art MRI-based attenuation correction was derived from T1-weighted MRI (pseudo-CT [pCT]). For validation purposes, a low-dose CT scan was also performed. Arterial blood samples were collected as the reference standard (arterial input function [AIF]). The developed pipeline allows the derivation of an image-derived input function (IDIF), which is subsequently used to create CMRGlc maps by means of a Patlak analysis. The pipeline also includes motion correction using the MRI navigator sequence as well as a novel partial-volume correction that accounts for background heterogeneity. Finally, CMRGlc maps are used to generate a normative database to facilitate the detection of metabolic abnormalities in future patient scans. To assess the performance of the developed pipeline, IDIFs extracted by both CT-based attenuation correction (CT-IDIF) and MRI-based attenuation correction (pCT-IDIF) were compared with the reference standard (AIF) using the absolute percentage difference between the areas under the curves as well as the absolute percentage difference in regional CMRGlc values. **Results:** The absolute percentage differences between the areas under the curves for CT-IDIF and pCT-IDIF were determined to be 1.4% ± 1.0% and 3.4% ± 2.6%, respectively. The absolute percentage difference in regional CMRGlc values based on CT-IDIF and pCT-IDIF differed by less than 6% from the reference values obtained from the AIF. **Conclusion:** By taking advantage of the capabilities of fully integrated PET/MRI, we developed a fully automated computational pipeline that allows the noninvasive determination of regional CMRGlc values in a clinical setting. This methodology might facilitate the proliferation of fully quantitative imaging into the clinical arena and, as a result, might contribute to improved diagnostic efficacy.

The prospect of deriving fully quantitative physiologic measurements of the human body that go beyond the differential evaluation of image patterns is a key strength of PET imaging (1). Moreover, the quantitative assessment of physiologic processes in vivo, such as metabolism, perfusion, or neurotransmitter receptor binding, is considered a necessary next step on the path to human precision medicine (2).

Pioneering studies in the early days of PET imaging clearly demonstrated the potential of absolute quantification in understanding human physiology (1). However, because of the complexity and invasiveness (i.e., arterial cannulation) of the protocols, their adoption into clinical work was limited. Instead, absolute quantification was supplanted in due course by less physiologic, but more practical, measures, such as SUVs (3). However, despite their indisputable clinical usefulness, such semiquantitative measures cannot provide information about underlying physiologic mechanisms and are meaningful only within the context of the diagnostic application.

To obviate the need for the arterial input function (AIF) in brain studies, several methodologies have been proposed to extract an image-derived input function (IDIF) directly from PET images (4–12). These studies demonstrated that the extraction of an IDIF from a dynamic PET dataset requires 3 main tasks: the accurate definition of a blood-pool region, the accurate correction for subject motion, and the exact correction of extracted time–activity curves for partial-volume effects because of the small diameter of the internal carotid arteries.

In past implementations, an IDIF was extracted either from PET/CT data in conjunction with a separate MRI scan (4,6,10,12) or from a fully integrated PET/MRI protocol (7,8,11,13). In particular, taking advantage of methodologic advances provided by fully integrated PET/MRI allows the 3 challenges mentioned earlier to be addressed in a straightforward manner.

For example, high-resolution MR sequences, such as 3-dimensional (3D) time-of-flight MR angiography (MRA), can be effectively used to localize the carotid vasculature, and the combined anatomic (vessel geometry) and physiologic (vessel tracer concentration) information can be used for the accurate correction of partial-volume (PV) distortions in the PET data. In addition, high-speed MRI navigators can be interleaved with clinical MR sequences to define motion vectors that allow the correction of subject motion (14).

PET/MRI-based IDIF methodologies have well-defined advantages over combined PET/CT and MRI protocols; however, they have suboptimal MRI-based attenuation correction (AC) that typically mandates a separate low-dose CT scan to derive a CT-based attenuation correction (CT-AC) map. Therefore, PET/MRI-based IDIF methodologies have been restricted to research because of their dependence on external CT scans and substantial postprocessing analysis schemes to extract an accurate IDIF.

The adoption of a noninvasive absolute quantification scheme in clinical routines requires both an automated analysis approach and independence from additional procedures (CT imaging). Here, we introduce a fully automated processing pipeline that enables the noninvasive absolute quantification of the cerebral metabolic rate for glucose (CMRGlc) in a clinical setting on the basis of synergistic information obtained from a fully integrated PET/MRI system. In this extension of our previous work (11), we now introduce MRI navigator–based motion correction that, apart from PET data alignment, also aligns the attenuation maps (dynamic AC maps) with PET emission data. Moreover, we now use an improved partial-volume correction (PVC) method that is sensitive to the spatial and temporal variations of the target and background activities. In addition, we have implemented a state-of-the-art MRI-based AC method (15) that abrogates the need for an external CT-AC map. Furthermore, we have incorporated an absolute quantification component that allows the calculation of CMRGlc maps using Patlak analysis and that also generates a normative database (NDB) that can be used to objectively define patient-specific brain abnormalities and directly support clinical readings. Finally, we have integrated all of these methodologic advances into a single automated processing pipeline, affording noninvasive absolute quantification in a clinical setting.

## MATERIALS AND METHODS

Ten healthy volunteers (5 men and 5 women; 27 ± 7 y old) were included in this study. The study was approved by the Ethics Committee of the Medical University of Vienna and was performed in accordance with the Declaration of Helsinki (1964), including current revisions. All volunteers were deemed to be healthy on the basis of their medical history, physical examinations, and vital signs. Written informed consent was obtained from all of the subjects before the examinations.

### Study Design

The subjects underwent test-retest examinations (mean time difference, 17 ± 44 d) in a fully integrated PET/MRI system (Biograph mMR; Siemens). To validate the accuracy of the IDIF, arterial blood samples were obtained from a radial artery. Moreover, after the PET/MRI examinations, a low-dose CT scan of the brain (120 kVp; 50 mAs) was acquired only for test examinations using a PET/CT system on-site (Biograph TruePoint TrueView 64; Siemens Healthineers) to compare the performance of MRI-based AC with that of CT-AC.

### Imaging Protocol

All examinations were conducted in the afternoon; subjects were asked to keep their eyes open without performing any task. Before each scan, the glucose concentration (mmol/L) in blood was measured, and a venous line was established for the injection of the ^18^F-FDG tracer. In addition, an arterial line was established in the contralateral arm for manual arterial blood sampling. To ensure a high signal-to-noise ratio in the MR images, a head and neck coil was used.

After the brain was positioned in the center of the field of view, a 60-min PET list-mode acquisition was initiated with the intravenous injection of ^18^F-FDG (352 ± 66 MBq), which was administered as a slow bolus over 40 s. Contemporaneously with the PET data acquisition, multiple MRI sequences were acquired: a 3D time-of-flight MR angiography (TOF-MRA) sequence (voxel size, 0.5 × 0.5 × 1 mm; echo time, 3.6 ms; repetition time, 21 ms; flip angle, 25°; matrix, 228 × 384; 220 slices**)** for the definition of the carotid vasculature, a T1-weighted MRI sequence (voxel size, 1 × 1 × 1 mm; matrix, 256 × 256; 192 slices) for the anatomic localization and calculation of the pseudo-CT (pCT) AC (pCT-AC) map, and a conventional Dixon sequence (voxel size, 2.60417 × 2.60417 × 3.12 mm; matrix, 192 × 128; 126 slices) for the generation of a Dixon AC map (16). In addition, sparsely sampled MRI navigators (2-dimensional echo-planar imaging; voxel size, 3.0 × 3.0 × 3.0 mm; matrix, 64 × 64; 36 slices; echo time, 30 ms; repetition time, 3,000 ms**)** were acquired for motion correction. MRI navigators were interleaved at specific time intervals (0, 2.5, 5, 7.5, 10, 14, 17, 21, 26, 33, 38, 42, 44, and 50.5 min after injection).

PET list-mode data were rebinned into a dynamic frame sequence (24 × 5 s, 1 × 60 s, 1 × 120 s, and 11 × 300 s) and reconstructed (Siemens e7 tools) into a 344 × 344 × 127 matrix (voxel size, 2.08 × 2.08 × 2.03 mm) using the ordinary Poisson ordered-subset expectation-maximization 3D algorithm (3 iterations, 28 subsets, and 2-mm gaussian filter). Attenuation and scatter correction were performed using AC maps corrected for motion.

### Blood Sampling

Arterial blood samples were collected manually from the radial artery at different time points (24 × 5 s, 1 × 60 s, 1 × 120 s, 1 × 300 s, 1 × 600 s, and 2 × 1200 s after injection). The blood sampling was performed using vacuum test tubes via an arterial cannula fitted with an adapter. Before every arterial sample was collected 2 min after injection, the line was flushed with 5 mL of sodium chloride solution to prevent clotting and sampling of stagnant blood. To avoid dilution of the actual sample, 1 mL of blood was drawn and discarded before the arterial blood sample was drawn. Whole-blood concentrations were measured using a γ-counter (2480 WIZARD^2^ automatic γ-counter; PerkinElmer). To obtain the AIF, whole-blood samples were centrifuged to separate the plasma component before the radioactivity in the plasma was measured. The measured whole-blood and plasma tracer concentrations were used to calculate the plasma-to-blood ratio for each subject.

### Attenuation Map Processing

The Dixon AC map and the first MRI navigator (Nav-0) were considered to be coregistered because they were acquired sequentially with a negligible temporal gap. To generate dynamic AC maps that considered patient motion (see additional information later in the article), the low-dose CT image volume and the T1-weighted MR image (acquired 10 min after the start of the PET acquisition) were aligned with the Dixon AC map as follows. Initially, a Dixon composite image was derived by summing the in-phase and out-of-phase fat and water images. This Dixon composite image volume served as the reference volume to which the CT image volume was rigidly aligned using SPM12 (Wellcome Trust Center for Neuroimaging, University College London) (17). After automatic removal of the CT bed, tube voltage–dependent bilinear scaling was applied to convert the low-dose CT image to a CT-AC map (18). In addition, a state-of-the-art MRI-based AC map (pCT map) was derived directly from T1-weighted MR images through a multiatlas propagation scheme, which locally matches the MRI-derived morphology to a database of MRI-CT pairs using a local image similarity measure (15). The obtained pCT map was rigidly coregistered to the low-dose CT map, and then tube voltage–dependent bilinear scaling (18) was applied to generate a pCT-AC map. CT-AC and pCT-AC maps were smoothed using Siemens e7 tools to match the spatial resolution of PET images.

### Noninvasive Quantification Pipeline

**FIGURE 1.**
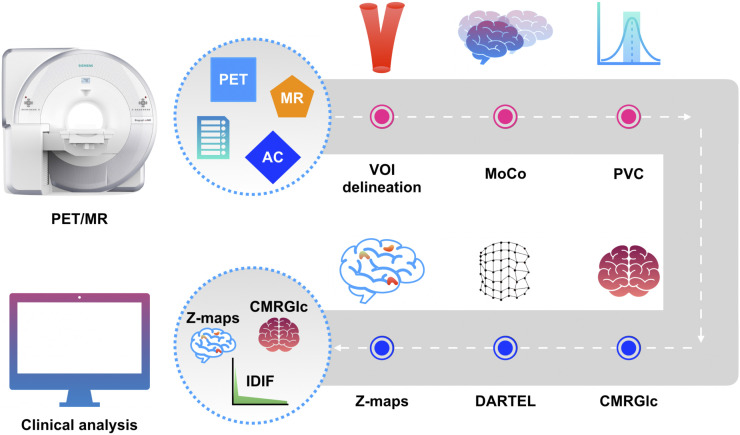
Noninvasive absolute quantification pipeline consisting of 6 nodes (depicted as circles); pink nodes correspond to IDIF-generating components, and blue nodes correspond to quantification components. Input consists of synergistic data from PET/MRI study along with parameter file, with output yielding IDIF as well as CMRGlc and abnormality maps (Z-maps). MoCo = motion correction; PVC = partial-volume correction; VOI = volume of interest.

The developed pipeline consists of an IDIF component and a quantification component (Fig. 1). Six nodes each perform a specific task in sequential order (Supplemental Fig. 1) (supplemental materials are available at http://jnm.snmjournals.org). The IDIF component performs dynamic PET reconstruction (Siemens e7 tools); this step is followed by the generation of an IDIF through the automatic delineation of a suitable volume of interest and the application of corrections for subject motion and partial-volume distortions. The IDIF is then forwarded to the quantification component, which creates a pixel-by-pixel CMRGlc map using Patlak analysis (19). The resulting CMRGlc maps are spatially normalized and incorporated into an NDB that can be subsequently used to create abnormality maps for individual patients.

### Automated Internal Carotid Artery (ICA) Segmentation

The petrous segment of the internal carotid artery (ICA) was chosen as the volume of the interest to extract the IDIF. The petrous region was automatically segmented from the 3D TOF-MRA sequence using an algorithm proposed by Sundar et al. (11). A combination of histogram-based quantile thresholding (20) and an automatic seeded region–growing algorithm created a mask of carotid vessels. Given that the petrous segment of the ICA was well defined by a distinct morphology (geometric orientation, axis length, and ellipticity), it could be easily extracted from axial slices, yielding the ICA target region (P_mask_).

### MRI-Driven Motion Correction

**FIGURE 2.**
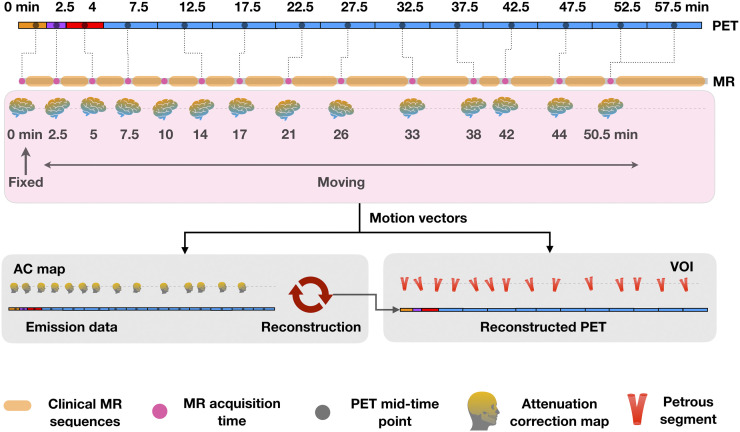
MR-driven motion correction as implemented in developed pipeline. MRI navigators are assigned to each PET frame on basis of smallest temporal difference, and obtained motion vectors are used for aligning both AC map and petrous volume of interest to PET image data. VOI = volume of interest.

Sparsely sampled MRI navigators interleaved between MR sequences were used to perform motion correction of PET images (14). The initial navigator (Nav-0) was considered to be the reference volume, and all subsequent navigators (Nav-1–Nav-13) were rigidly aligned to Nav-0 using SPM12, yielding a set of motion vectors (MV-1–MV-13; 3 translations and 3 rotation parameters). Correspondence between the MRI navigators and PET emission data was assumed on the basis of the smallest temporal difference between the respective MRI navigator acquisition time and the midscan time of the PET frame (Fig. 2).

To account for spatial misalignment between the initially determined static AC map and the PET emission data, the inverse of the motion vectors was applied to this AC map (CT or pCT), resulting in a set of motion-corrected AC maps. The obtained motion-corrected AC maps were then used for reconstruction of the dynamic PET emission data using Siemens e7 tools. Navigators were also used to account for the misalignment between the P_mask_ and the dynamic PET frames. Because the TOF-MRA sequence was acquired at the start of the PET acquisition (sequentially with the Dixon AC map and Nav-0), we assumed these image volumes to be aligned. All subsequent coregistrations were visually confirmed using AMIDE 1.0.5 software (21).

### PVC

PVC was performed using a modified version of the Müller-Gärtner algorithm (22), also taking into account the radial, circumferential, and temporal variabilities of the background activity surrounding the P_mask_. PVC entails both spill-in and spill-out corrections, for which knowledge of the point spread function (PSF) of the PET system is required. We previously determined the PSF of the PET/MRI system used here as a 3D gaussian function with an isotropic full width at half maximum of 6.0 mm (11).

**FIGURE 3.**
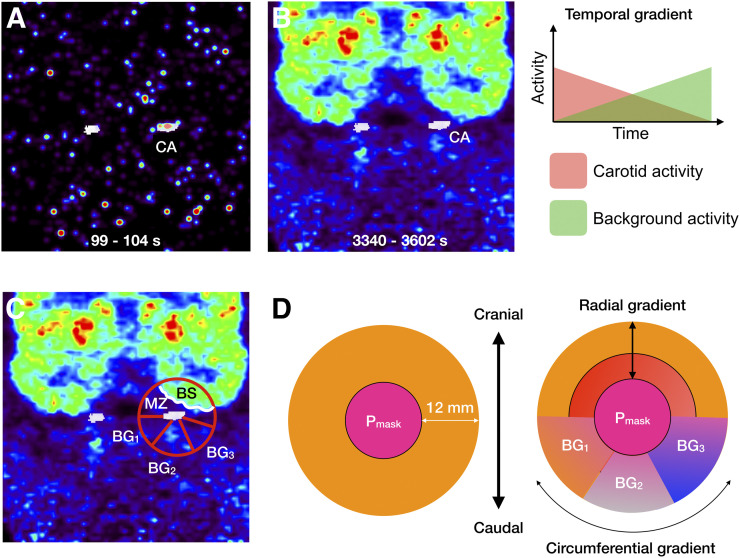
(A and B) PET frame reconstruction with ICA overlay (white) for early (A) and late (B) times after injection. Temporal and spatial variabilities of ICA background can be clearly deduced from images. (C) Tracer distribution in vicinity of ICA displays both radial and circumferential tracer concentration gradients. (D) Definition of subregions in vicinity of P_mask_ used to account for partial-volume distortions. BG_1,_ BG_2_, and BG_3_ = various background regions with homogeneous tracer concentrations; BS = brain activity; CA = measured activity in ICA; MZ = activity in MZ.

An accurate correction for partial-volume distortions that affected the apparent tracer concentration in the P_mask_ mandated correction for background heterogeneity in both the radial and the circumferential directions (Fig. 3). To accomplish such a correction, a background mantel was initially created by dilating the P_mask_ from the edge by 5 voxels (∼12 mm, equivalent to 2 times the full width at half maximum) to mark the region from which the tracer concentration potentially spilled over into the P_mask_. At late time points, a strong radial tracer concentration gradient was determined toward the brain, whereas the tracer gradient toward soft tissue in the neck was found to exist predominantly in the circumferential direction (Fig. 3).

To determine the location of brain tissue within the background mantel, a brain mask was derived from the T1-weighted MR image volume using the SPM12 segmentation algorithm. The overlap between the background mantel and the brain mask represented the brain segment (BS) that contributed to partial-volume distortions in the P_mask_. Moreover, the region located between the BS and the P_mask_ represented the mixed zone (MZ), as it received contributions from both the BS and the P_mask_. Segmentation of the tracer concentration using Otsu thresholding (23) at each time frame resulted in the following volumes within the background mantel: brain tissue (BS^vol^); the region located between the BS and the P_mask_, representing the MZ (MZ^vol^); and background sections (BG^vol^_j_, where j – 1,...M), representing heterogeneous tracer concentrations expressed predominantly in the circumferential direction (Fig. 3). All volumes contributed independently to partial-volume distortions in the P_mask_ (CA^vol^), resulting in the measured arterial tracer concentration CA.

The corresponding measured tracer concentrations were denoted as CA, BS, MZ, and BG_j_ (measured tracer concentration in the j-th BG segment), and the true (partial-volume–corrected) tracer concentrations for each of the subvolumes were denoted as CA′, BS′, MZ′, and BG′_j_, respectively. Before all calculations, the subvolumes were corrected for subject motion using the inverse of the motion vectors. To correct for the bidirectional spillover between subvolumes, an iterative approach for estimating the true tracer concentration in the ICA target volume (CA′) was applied. On the basis of the model shown in Figure 3, CA′ can be expressed as

Eq. 1 $$\begin{array}{c} \text{CA}\prime =\frac{1}{{\text{RCA}}_{\text{CA}}}\left(\text{CA}-\text{BS}\prime \;\times \;{\text{RBS}}_{\text{CA}}-\text{MZ}\prime \;\times \;\text{RM}{\text{Z}}_{\text{CA}}-\sum_{\text{j}=1}^{\text{M}}{\text{BG}}_{\text{j}}\prime \;\times \;{\text{RBG}}_{\text{jCA}}\right) \end{array},$$

where the geometric factors RCA_CA_, RBS_CA_, RMZ_CA_, and RBG_jCA_ represent the convolution of the known PSF and the individual volumes CA^vol^, BS^vol^, MZ^vol^, and BG^vol^_j_, respectively, averaged at the location of CA^vol^ (the symbol ⊗ represents the convolution operation):

Eq. 2 $$\begin{array}{c} {\text{RCA}}_{\text{CA}}={\left({\text{P}}_{\text{mask}}⊗\text{PSF}\right)}_{{\text{CA}}^{\text{vol}}} \end{array},$$

Eq. 3 $$\begin{array}{c} {\text{RBS}}_{\text{CA}}={\left({\text{BS}}^{\text{vol}}⊗\text{PSF}\right)}_{{\text{CA}}^{\text{vol}}} \end{array},$$

Eq. 4 $$\begin{array}{c} {\text{RMZ}}_{\text{CA}}={\left({\text{MZ}}^{\text{vol}}⊗\text{PSF}\right)}_{{\text{CA}}^{\text{vol}}} \end{array},$$

Eq. 5 $$\begin{array}{c} {\text{RBG}}_{\text{jCA}}={\left({\text{BG}}_{\text{j}}^{\text{vol}}⊗\text{PSF}\right)}_{{\text{CA}}^{\text{vol}}} \end{array}.$$

On a conceptual level, Equation 1 implies that the true arterial concentration in the target region (CA′) can be calculated by first correcting the measured arterial concentration (CA) for spillover from the true tracer concentrations in the brain (BS′), the MZ (MZ′), and the various background segments (BG′_j_) and then applying the spill-out correction for the target region (RCA_CA_). However, the true tracer concentrations BS′, MZ′, and BG′_j_ are unknown because the measured concentrations BS, MZ, and BG_j_, respectively, include an unknown spillover component from CA′.

Thus, an iterative process is initiated by assuming the spillover contribution from CA′ to the neighboring tissues to be negligible, so that BS′ = BS, MZ′ = MZ, and BG′_j_ = BGj. Using these assumptions, an initial estimate of CA′ (CA′_0_) can be calculated as

Eq. 6 $$\begin{array}{c} {\text{CA}}_{0}\prime =\frac{1}{{\text{RCA}}_{\text{CA}}}(\text{CA}-\text{BS}\;\times \;{\text{RBS}}_{\text{CA}}-\text{MZ}\;\times \;{\text{RMZ}}_{\text{CA}}-\sum_{\text{j}=1}^{\text{M}}{\text{BG}}_{\text{j}}\;\times \;{\text{RBG}}_{\text{jCA}}. \end{array}$$

Once an estimate of CA′ has been calculated (CA′_n_), it can be iteratively improved by recalculating new estimates for BS′_n+1_, BG_j n+1_′, and MZ′_n+1_, yielding the updated CA′_n+1_, as follows:

Eq. 7 $$\begin{array}{c} {\text{BS}}_{\text{n}+1}\prime =\frac{1}{{\text{RBS}}_{\text{BS}}}\left(\text{BS}-{\text{CA}}_{\text{n}}\prime \;\times \;{\text{RCA}}_{\text{BS}}\right) \end{array},$$

Eq. 8 $$\begin{array}{c} {\text{BG}}_{\text{jn}+1}\prime =\frac{1}{{\text{RBS}}_{\text{jBGj}}}\left(\text{BS}-{\text{CA}}_{\text{n}}\prime \;\times \;{\text{RCA}}_{\text{BGj}}\right) \end{array},$$

Eq. 9 $$\begin{array}{c} {\text{MZ}}_{\text{n}+1}\prime =\frac{1}{{\text{RMZ}}_{\text{MZ}}}\left(\text{MZ}-{\text{CA}}_{\text{n}}\prime \;\times \;{\text{RCA}}_{\text{MZ}}-{\text{BS}}_{\text{n}+1}\prime \;\times \;{\text{RBS}}_{\text{MZ}}-\sum_{\text{j}=1}^{\text{M}}{\text{BG}}_{\text{j}.\text{n}+1}\prime \;\times \;{\text{RBG}}_{\text{jMZ}}\right) \end{array},$$

Eq. 10 $$\begin{array}{c} {\text{CA}}_{\text{n}+1}\prime =\frac{1}{{\text{RCA}}_{\text{CA}}}\left(\text{CA}-{\text{BS}}_{\text{n}+1}\prime \;\times \;{\text{RBS}}_{\text{CA}}-{\text{MZ}}_{\text{n}+1}\prime \;\times \;{\text{RMZ}}_{\text{CA}}-\sum_{\text{j}=1}^{\text{M}}{\text{BG}}_{\text{j}.\text{n}+1}\prime \;\times \;{\text{RBG}}_{\text{jCA}}\right) \end{array}.$$

This iterative procedure is terminated once the difference between successive values for CA′ achieves convergence.

An important observation is that during the first pass, the tracer is predominantly present in the arteries and the spill-in contribution from the background region is negligible because there is no uptake in the surrounding tissues (Fig. 3). Therefore, Equation 1 can be simplified to

Eq. 11 $$\begin{array}{c} \text{CA}\prime =\frac{1}{{\text{RCA}}_{\text{CA}}}\left(\text{CA}\right) \end{array}.$$

Two assumptions are inherent in our model. First, the contribution of MZ to BS is negligible, given that the uptake of ^18^F-FDG in brain tissue is either as low as or much greater than that in the connective tissue of the neck. Second, the spillover components among the background regions (BG_j_) can be reasonably ignored in light of the circumferential tracer concentration gradient in neck tissue being a relatively slowly changing function, thus rendering spillover effects within the background regions of minor importance.

### Postprocessing of IDIF

After motion correction and PVC, the IDIF was interpolated with a step length of 1 using a piecewise cubic Hermite interpolating polynomial to match the blood sampling times. All corrections were applied to the IDIF because the AIF is considered to be the reference standard (10,11). First, the counts per minute (cpm) obtained from sampled arterial blood were scaled using the cross-calibration factor (kBq/cm^3^/cpm) between the PET/MRI system and the on-site γ-counter. Second, a plasma IDIF was based on the individual plasma-to-blood ratios obtained from the sampled arterial blood from the study subjects. Third, the delay between the AIF and the IDIF was corrected by shifting the IDIF curve to match the arrival times of the AIF. Finally, because of the difference in sampling locations (ICA for the IDIF and radial arteries for the AIF), a monoexponential dispersion function with a tau value of 5 s (24,25) was convolved with the IDIF to mimic the dispersion effects. The procedure was repeated for CT-AC–based PET images and pCT-AC–based PET images to derive their respective IDIFs (CT-IDIF and pCT-IDIF).

### Quantification (Patlak Graphical Analysis)

Motion vectors derived from the MRI navigators were applied to the corresponding PET frames, resulting in motion-corrected PET frames. After the spatial alignment, a voxelwise Patlak graphical analysis (lumped constant, 0.65 (26)) was performed using time–activity curves derived from motion-corrected PET frames in combination with their respective AIFs and IDIFs. Analysis was performed using an in-house–developed MATLAB tool (MATLAB R2018a; The MathWorks, Inc.) that generated parametric images representing CMRGlc (μmol/100 g/min). Specifically, a linear function was fitted to the Patlak graphical analysis–transformed data, including data from 25 min after injection until the end of the study (8 data points). The resulting slope was then multiplied by the subject’s plasma glucose level (μmol/L) and divided by the lumped constant.

### Assessment of NDB Generated from IDIF

For each subject, T1-weighted MR images were coregistered to their respective PET images. Individual T1-weighted MR image volumes were subsequently spatially normalized using the DARTEL (Diffeomorphic Anatomic Registration Through Exponentiated Lie algebra) software implemented in SPM12. The resulting deformation fields were then applied to the coregistered CMRGlc images, yielding a set of CMRGlc images that were transformed into template space.

Once in template space, the set of CMRGlc images (*n* = 20) defined mean (μ) and SD (σ) maps that constituted the NDB. Multiple NDBs (μ and σ images) were created separately from CMRGlc images derived using the AIF (NDB_AIF_: μ_AIF-CT_, σ_AIF-CT_), CT-IDIF (NDB_CT-IDIF_: μ_CT-IDIF_, σ_CT-IDIF_), and pCT-IDIF (NDB_pCT-IDIF_: μ_pCT-IDIF_, σ_pCT-IDIF_).

### Quantitative Comparison of Input Functions and CMRGlc Values

The areas under the curves (AUCs) of the 3 input functions (AIF, CT-IDIF, pCT-IDIF) were quantitatively compared using the absolute percentage error: |%Δ| = [(AUC for IDIF − AUC for AIF)/AUC for AIF] × 100. Moreover, test-retest variability (Var) with respect to both AUCs and CMRGlc values was assessed as

Eq. 12 $$\begin{array}{c} \text{Var}=\left(\frac{\left|\text{Test}-\text{Retest}\right|}{\frac{\text{Test}+\text{Retest}}{2}}\right)\times 100. \end{array}$$

Finally, to assess differences in regional CMRGlc values determined on the basis of the IDIFs against the AIF, the absolute percentage error between CMRGlc values based on the 3 input functions was calculated for 6 brain regions: corpus callosum, brain stem, cerebellum, thalamus, anterior cingulate cortex, and superior frontal cortex. Standardized regions based on the Hammersmith atlas (27) were applied in the NDBs, yielding values for μ and σ images for each anatomic region across the normal population.

## RESULTS

**FIGURE 4.**
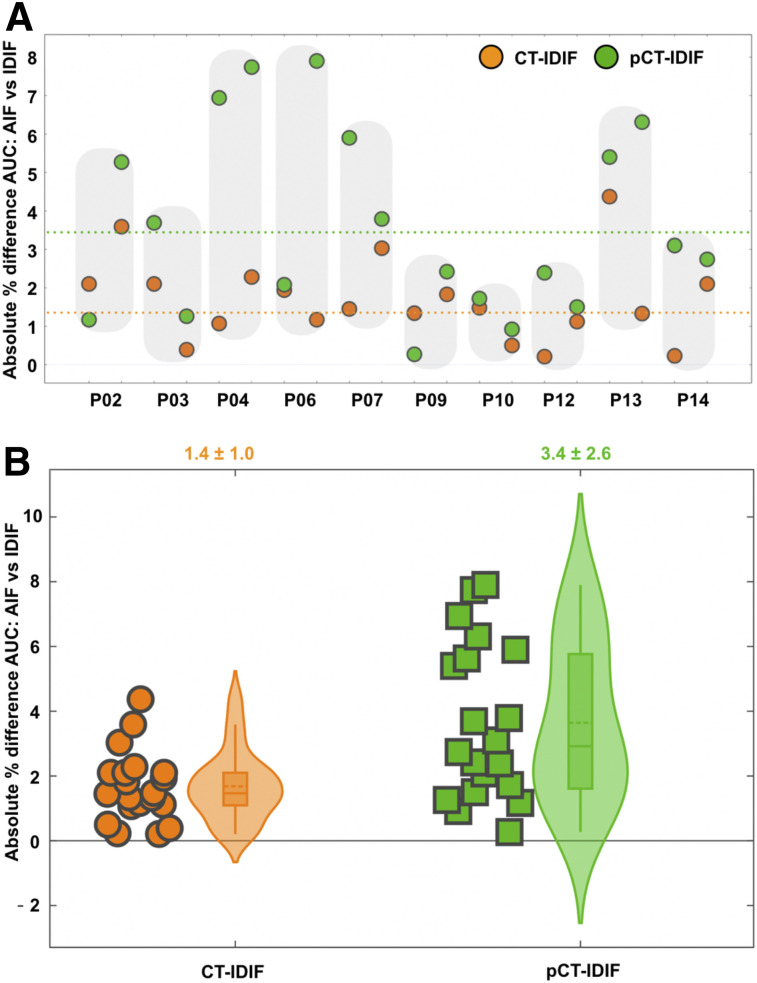
Comparison of IDIFs using AUCs. (A) Individual absolute percentage differences in AUCs for CT-IDIF and pCT-IDIF against AIF. Shaded areas indicate test-retest results for same subject. Broken lines represent mean difference over all scans between AUCs derived using AIF and CT-IDIF (orange) and those derived using AIF and pCT-IDIF (green). (B) Plot of absolute percentage differences in AUCs for AIF and IDIFs (CT-IDIF and pCT-IDIF). Shaded area enclosing box plot indicates probability density distribution for absolute percentage differences. Average absolute percentage difference for both methods was <5% (shown above graph).

Figure 4 shows a comparison of input functions based on CT-AC (CT-IDIF) and pCT-AC (pCT-IDIF) against the reference standard of arterially sampled blood (AIF). The AUC derived using the CT-IDIF showed excellent agreement with the AIF, with an absolute percentage error of 1.4% ± 1.0%. In contrast, the AUC obtained using the pCT-IDIF showed higher variability, with an absolute percentage error of 3.4% ± 2.6% against the AIF-derived AUC. Sample input function curves are shown in Supplemental Figure 2.

**FIGURE 5.**
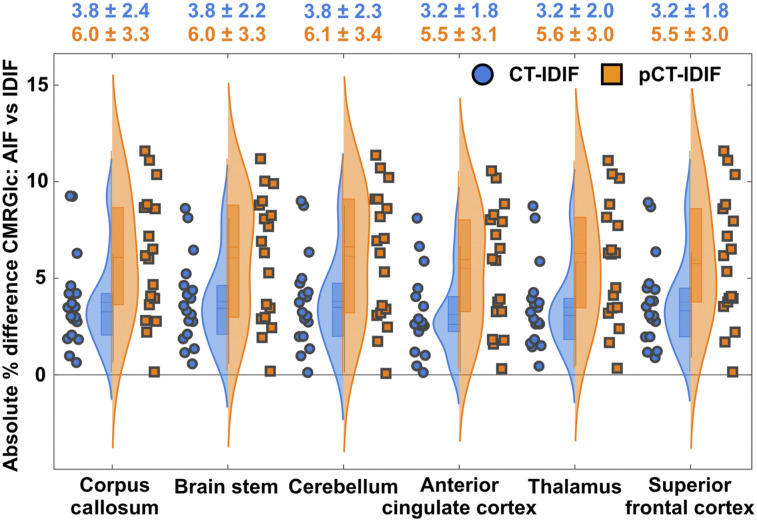
Probability density distribution for absolute percentage differences between CMRGlc values derived using AIF and those derived using IDIFs (CT-IDIF and pCT-IDIF) for 6 different brain regions. Absolute percentage differences in CMRGlc values derived using CT-AC are shown in blue, and those derived using pCT-AC are shown in orange. Mean and SD for each region and 2 AC methods are shown above graph.

A comparison of regional CMRGlc values demonstrated excellent agreement between values derived using the AIF and image-derived values (Fig. 5). The absolute percentage error of regional CMRGlc values associated with the pCT-IDIF (5.8% ± 3.2%) was higher than that associated with the CT-IDIF (3.5% ± 2.1%).

The difference in whole-brain CMRGlc values between test and retest scans was found to be much greater than the difference between the individual methods for extracting an IDIF. The absolute percentage errors between repeated whole-brain CMRGlc values across the group were 13.3% ± 10.0% for the AIF and 13.0% ± 11.0% and 17.6% ± 15.0% for CMRGlc values determined using the CT-IDIF and pCT-IDIF, respectively (Supplemental Fig. 3). Regional μ and σ values obtained from all scans are shown in Table 1. The data indicated a coefficient of variation (COV) [(σ/μ) × 100%] of 19% ± 3% for regional CMRGlc values derived using the pCT-IDIF. The COV for CMRGlc values derived using the AIF was determined to be 18% ± 4%—similar to the COV derived using the CT-IDIF (18% ± 3%).

**TABLE 1:** Regional CMRGlc Values in NDB for 6 Reference Regions in Brain

	CMRGlc values* obtained from:					
	AIF		CT-IDIF		pCT-IDIF	
Region	Mean ± SD	COV (%)	Mean ± SD	COV (%)	Mean ± SD	COV (%)
Corpus callosum	16.1 ± 3.6	22	16.1 ± 3.6	22	15.9 ± 4.0	25
Brain stem	20.0 ± 2.7	14	19.9 ± 2.7	14	19.6 ± 3.1	16
Cerebellum	24.6 ± 3.4	14	24.6 ± 3.5	14	24.3 ± 4.1	17
Anterior cingulate	31.9 ± 6.4	21	31.8 ± 6.3	20	31.4 ± 7.0	22
Thalamus	34.3 ± 5.9	17	34.2 ± 5.8	17	33.7 ± 6.6	20
Superior frontal	34.4 ± 6.6	19	34.2 ± 6.6	19	33.8 ± 7.3	22

* Reported as μmol/100 g/min.

Maximum deviations from AIF standard of CMRGlc obtained from CT-IDIF and fully automated pCT-IDIF were 10% and 12%, respectively.

**FIGURE 6.**
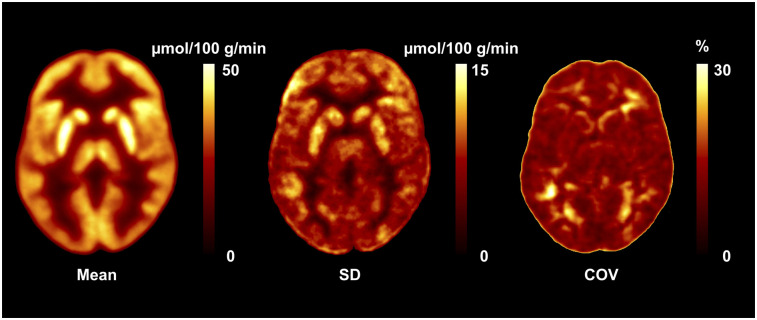
Database images in Montreal Neurological Institute space representing mean, SD, and COV maps for absolute values of CMRGlc. COV map indicates normal physiologic variability of 15%–25%.

Figure 6 shows the μ, σ, and COV images obtained from CMRGlc values derived from an IDIF based on pCT-AC. Because of the physiologic variation in CMRGlc values, the normal COV across the brain ranged from 15% to 25% (Supplemental Fig. 4).

## DISCUSSION

We present here a fully automated approach that allows the noninvasive determination of CMRGlc maps using dynamic ^18^F-FDG imaging with a fully integrated PET/MRI system. Our objective was to develop a methodology that allows the clinical quantification of ^18^F-FDG PET data in addition to complementary visual information. Such readings are markedly influenced by the assessment of tracer uptake asymmetries. As a result, expert interpreters often find themselves in a situation in which they have to make a judgment call about the clinical value of a particular asymmetry. The role of quantitative PET analysis is to provide added information with respect to the identification of suspicious territories that warrant a closer inspection by the clinical interpreter. Such an approach could provide greater confidence in the clinical interpretation of PET scans.

Previous attempts directed toward using PET/MRI methodology for quantitative imaging based on an IDIF limited the use of MRI information to the definition of arterial target regions and correction for partial-volume distortions. For example, Jochimsen et al. (7) used structural information from MRI to apply PVC based on a geometric transfer matrix (28) approach to extract the true arterial tracer concentration. Although the geometric transfer matrix provides a closed solution to this problem, it requires accurate structural parcellations of both the arterial target region and the various (physiologically heterogeneous) surrounding tissues. However, this information is extremely difficult to obtain from MRI segmentation. Sari et al. (10) proposed a less demanding alternative, the single-target correction PVC, which requires only the segmentation of the arterial target region. However, the single-target correction PVC is highly dependent on both the accurate alignment of PET and MRI data and the exact estimation of the PSF of the PET system. In addition to being highly sensitive to motion artifacts, both of these methods inherently assume that the tracer concentration distribution (and the resulting parcellation) varies spatially, but not temporally (4–12). However, the importance of considering the temporal variability of the target and background regions during IDIF extraction was emphasized previously (29).

In our current implementation, we took advantage of the synergistic “anatometabolic” information obtained from the fully integrated PET/MRI protocol to account for both the dynamic radial and circumferential variability of the background regions and to use sparsely sampled MRI navigators to correct for subject motion in both emission and transmission spaces. Moreover, to obviate the need for acquiring an additional CT scan, which was a key limitation of our prior work (11), we implemented and validated a state-of-the-art MRI-based AC (pCT (15)). Our results indicated that although the IDIF based on pCT-AC resulted in higher variance in CMRGlc maps than the IDIF based on CT-AC, the absolute percentage difference remained within 6% of CMRGlc values derived using the reference standard of arterially sampled blood and CT-AC (Table 1). Accordingly, we suggest that pCT-AC is acceptable for clinical work because of its feasibility, robustness (30), and availability.

Taken together, our results demonstrated the feasibility of quantification of the absolute metabolic rate of glucose in the brain using combined PET/MRI, without the need for arterial blood sampling or the need for an additional low-dose CT brain scan for AC. Specifically, our data showed excellent agreement between regional CMRGlc values calculated on the basis of arterial blood sampling and those determined using an IDIF that was corrected for attenuation using an MRI-derived pCT attenuation map. Nevertheless, our results also indicated that the MRI-derived pCT attenuation map was still inferior to the CT-derived attenuation map at the base of the skull—the area from which the IDIF was extracted. Consequently, further studies that could provide better AC maps derived from MR images at the base of the skull and neck regions are warranted (30).

Further, the derivation of an IDIF requires the use of a suitable anatomic region that can be reliably defined using an automated approach and that aids in the application of an accurate PVC. On the basis of their locations in the field of view of the PET/MRI system and their relatively large diameters, the cervical and petrous segments of the ICA appear to be well suited for the extraction of an IDIF. Although the cervical segments of the ICA feature a sufficiently large diameter (>5 mm), they are also subject to considerable physiologic variation, deviating in approximately 25% of subjects from a straight to a tortuous geometry (31). In contrast, the petrous segment of the ICA is a well-accepted landmark for neurosurgeons because of its 90° bend when entering the carotid canal (petrous angle) and is considered to be the most vital and easily visualized structure on MRA images (32). This distinct geometry allows reliable automated segmentation of the petrous ICA segment, although on rare occasions signal loss might occur at the genu of the petrous segment. Nevertheless, this segment of the ICA appears to be best suited for the extraction of an accurate IDIF.

Because the ICA diameter is in the same range as the full width at half maximum of the PET system, the determination of an IDIF is highly sensitive to local misregistration arising from involuntary patient motion (Supplemental Fig. 5). As such, accurate motion correction is mandatory for the extraction of an accurate IDIF (33). Here, our implementation included sparsely sampled MRI navigators that were acquired throughout the study, yielding a motion vector for every PET frame. In addition, inverted motion vectors were used to align both AC maps and regions of interest with the PET emission data, leaving the reconstructed PET images untouched. This approach prevented further smoothing of PET images caused by resampling, which would have negatively affected PVC. To assess the improvement in the accuracy of CMRGlc values due to the applied PVC, we performed a comparison of global CMRGlc values determined with and without PVC (using CT-AC) against the reference standard of AIF-derived global CMRGlc values. With the application of PVC, the absolute percentage difference between global CMRGlc values was reduced from 14.7% ± 7.6% to 3.7% ± 2.3%; these data support the efficacy of the implemented PVC.

At present, the developed pipeline has several limitations, which might forestall its rapid translation into clinical routines. For example, absolute quantification requires extended imaging times to measure the ^18^F-FDG uptake period in tissue (∼40 min). This duration is about twice that of a clinical static ^18^F-FDG brain scan. Thus, there is a cost involved in performing a quantitative PET scan; accordingly, quantification might not be appropriate for every patient in a clinical setting. Instead, we envision that quantitative scans will be reserved for selected patients—those for whom the knowledge of absolute CMRGlc values might provide added diagnostic value, such as patients with nonlesional extratemporal lobe epilepsy (34).

In addition, there are several methodologic issues that need to be considered. To successfully delineate the petrous ICA segment, the implemented algorithm requires specialized sequences, such as TOF-MRA, that are not necessarily routinely used in clinics. Such TOF-MRA sequences are poorly suited for patients with vessel stenosis because there is usually a signal drop in the clogged region. Another issue is motion artifacts. The quality of motion compensation schemes is crucial for the clinical implementation of our method, especially because the postprocessing motion compensation approach used is restricted to interframe motion correction without the ability to account for intraframe subject motion. Subjects in our study were specifically instructed to suppress movement during the scan; as a result, the observed motion magnitude was minimal (maximal translation of <4 mm; maximal rotation of <4° in all 3 axes). However, in clinical situations, the motion magnitude may be significantly higher because of patient noncompliance. Apart from these methodologic limitations, the usefulness of the pipeline is limited to tracers that have either no metabolite fraction or a known and stable plasma-to-blood ratio. In our previous study (11), we showed that the average plasma-to-blood ratio for ^18^F-FDG in the control group (*n* = 20) was 1.06 ± 0.01. Therefore, it is possible to convert blood IDIF to plasma IDIF without using any arterial blood samples in the case of ^18^F-FDG.

The fully quantitative assessment of CMRGlc provides valuable and detailed information about the regional metabolic state of brain tissue, but this advance in methodology brings its own set of issues that need to be carefully considered. Early studies investigating absolute CMRGlc in control subjects revealed a surprisingly large physiologic variability—in the range of 10%–20%—even for large regions and with the same subject being scanned only a few days (Supplemental Fig. 3) apart (35,36). Our own data confirmed these previous findings (Fig. 6). Consequently, in the absence of improved data acquisition protocols that are able to standardize the psychologic state of the subjects under study, the sensitivity for detecting areas of significantly increased or decreased CMRGlc will be relatively low, requiring about 25% deviation from the baseline. This sensitivity compares unfavorably with that of the visual assessment of regional asymmetries between homotopic brain areas, which can be quite easily detected at the 10%–15% level. Thus, to improve the relevance of absolute quantification for the detection of brain abnormalities, standardization of the subjects’ psychologic state will be necessary. Unfortunately, how such standardization could be achieved is currently unclear.

## CONCLUSION

We presented here a fully automated, clinically feasible PET/MRI processing pipeline that allows the generation of CMRGlc maps from dynamic ^18^F-FDG PET brain scans in a clinical setting. We hope that our work will aid in the proliferation of quantitative imaging into the clinical arena and, as such, will contribute to the ultimate goal of personalized imaging.

## DISCLOSURE

This work was supported by the Austrian Science Fund (KLI482-B31). No other potential conflict of interest relevant to this article was reported.

## KEY POINTS

**QUESTION:** Can a fully automated analysis pipeline for the noninvasive determination of the cerebral metabolic rate for glucose (CMRGlc) be established using anatometabolic information obtained from a fully integrated PET/MRI protocol?
**PERTINENT FINDINGS:** The developed approach allows the accurate noninvasive determination of an image-derived input function. The developed noninvasive method yields CMRGlc values that are within 6% of those determined using arterial sampling.
**IMPLICATIONS FOR PATIENT CARE:** Advances in the methodology of integrated PET/MRI allow the determination of absolute regional CMRGlc values in a clinical setting and therefore may provide added diagnostic value in patients with various neurologic diseases.
